# Green Synthesized Phytochemically (*Zingiber officinale* and *Allium sativum*) Reduced Nickel Oxide Nanoparticles Confirmed Bactericidal and Catalytic Potential

**DOI:** 10.1186/s11671-020-3283-5

**Published:** 2020-03-02

**Authors:** Ali Haider, Muhammad Ijaz, Sidra Ali, Junaid Haider, Muhammad Imran, Hamid Majeed, Iram Shahzadi, Muhammad Muddassir Ali, Jawaria Ali Khan, Muhammad Ikram

**Affiliations:** 1 Department of Clinical Medicine and Surgery, University of Veterinary and Animal Sciences, Lahore, Punjab 54000 Pakistan; 2 Department of Gynaecology& Obstetric (Unit –III), Jinnah Hospital, Lahore, Punjab 54000 Pakistan; 3 Tianjin Institute of Industrial Biotechnology, Chinese Academy of Sciences, 32 West 7th Avenue, Tianjin, 300308 China; 4 State key Laboratory of Chemical Resource Engineering, Beijing Advanced Innovation Centre for Soft Matter Science and Engineering, Beijing Engineering Center for Hierarchical Catalysts, Beijing University of Chemical Technology, Beijing, 100029 China; 5 Department of Food Sciences, Cholistan University of Veterinary and Animal Sciences, Near DHA663100, Bahawalpur, Pakistan; 6 University College of Pharmacy, University of the Punjab, Lahore, 54000 Pakistan; 7 Institute of Biochemistry and Biotechnology, University of Veterinary and animal sciences, Lahore, Punjab 54000 Pakistan; 8 Solar Cell Applications Research Lab, Department of Physics, Government College University, Lahore, Punjab 54000 Pakistan

**Keywords:** Metal oxide, Particle size, Diseases

## Abstract

Phyto-synthesized nanoparticles (NPs) having reduced chemical toxicity have been focused globally and become essential component of nanotechnology recently. We prepared green phytochemically (ginger and garlic) reduced NiO-NPs to replace synthetic bactericidal and catalytic agent in textile industry. NPs were characterized using ultra-violet visible spectroscopy (UV-Vis), X-ray diffraction (XRD), X-ray photoelectron spectroscopy (XPS), Fourier-transform infrared spectroscopy (FTIR), energy-dispersive X-ray spectroscopy (EDS), scanning electron microscopy (SEM), and transmission electron microscopy (TEM). The synthesis of NPs was confirmed by XRD and UV-Vis having strong absorption at 350 nm with size ranged between 16–52 nm for ginger and 11–59 nm for garlic. Scanning and transmission electron microscopy confirmed pleomorphism with cubic- and more spherical-shaped NPs. Moreover, exact quantities of garlic and ginger extracts (1:3.6 ml) incorporated to synthesize NiO-NPs have been successfully confirmed by FTIR. Phytochemically reduced NPs by garlic presented enhanced bactericidal activity against multiple drug-resistant *Staphylococcus aureus* at increasing concentrations (0.5, 1.0 mg/50 μl) and also degraded methylene blue (MB) dye efficiently. Conclusively, green synthesized NiO-NPs are impending activists to resolve drug resistance as well as environment friendly catalytic agent that may be opted at industrial scale.

## Introduction

Nanotechnology matter influence with at least one dimension size 1–100 nm that provides ability to engineer material by controlling their size [17]. NPs due to their unique chemical, physical, and biological properties in various fields, including medicine, have attained great attention. Their properties can be easily altered by changing size at nanometer scale [47].

Nickel (Ni) and nickel oxide (NiO) NPs have great importance due to their particular magnetic, catalytic, and electronic properties in energy technology, magnetism, biomedicines, and electronics [9, 26, 35]. NiO with a wide band gap of 3.6 to 4.0 eV and cubic lattice structure has potential due to p-type semiconductor. These NPs having high chemical stability, super capacitance properties, electron transfer capability, and electro catalysis are being used in biomedicines and photocatalytic, anti-inflammatory, and antibacterial activities [8, 10, 11, 45]. The emergence of infectious maladies, especially antibiotic-resistant (MDR), has devastated public health worldwide. Generally, both pathogenic Gram-positive (G +ve) and Gram-negative (G −ve) bacterial strains are among major public health threats.

In dairy industry, bovine mastitis is a major problematic disease having great economic impact characterized by chemical, microbiological, and physical changes in milk, while pathological changes in udder glandular tissues [6, 19]. Mastitis etiology includes infectious agents, i.e., bacteria, viruses, and fungi and most important are bacteria, divided into two groups: major (*Streptococci, Staphylococcus aureus*, *Corynebacterium pyogenes*, and *Coliform*) and minor pathogens (*Corynebacterium bovis* and *coagulase-negative Staphylococci*) [25]. The emergence of multiple drug-resistant Gram-positive and Gram-negative bacterial strains poses significant threat to public health [23, 32].

*Zingiber officinale* (ginger) is an important ingredient in Ayurvedia and Unani, and Chinese herbal medicine is treated for various ailments such as anti-nausea, digestive aid, rheumatism, and bleeding disorders due to wide diversity of volatile oils like zingiberol, monoterpene, sesquiterpene, and sesquiterpene hydrocarbons [12, 13, 43]. However, *Allium sativum* (garlic) contain organo-sulfur components, i.e., allyl sulphide groups, alliin, ajoene, allyl cysteine and allicin, and others such as vitamins, phospholipids, flavonoids, amino acids, and fatty acids that orient its medical properties [14, 24]. We aimed to assess bactericidal action of phytochemically reduced Ni metal oxide NPs against MDR (*S. aureus*), an isolate of bovine mastitis, and this will be the first report from Pakistan in veterinary research area on above mentioned agent.

## Methods

The current study was aimed at investigating the bactericidal action of phytochemically reduced NiO-NPs against MDR (*S. aureus*), an isolate of bovine mastitis.

### Materials

Nickel nitrate [Ni(NO_3_)_2_], sodium hydroxide (NaOH), methylene blue (MB), and sodium borohydride (NaBH_4_) of analytical grade were purchased from Sigma-Aldrich®, and fresh ginger and garlic roots were collected from the local market. Roots were dried in shade to achieve constant weight for further processing. Antibiotic discs were purchased from Bioanalyse® (Turkey). Bacterial growth media used were of analytical grade by TM Media, (Titan Biotech Ltd, India).

### Preparation of Aqueous Extracts

**Fig. 1:**
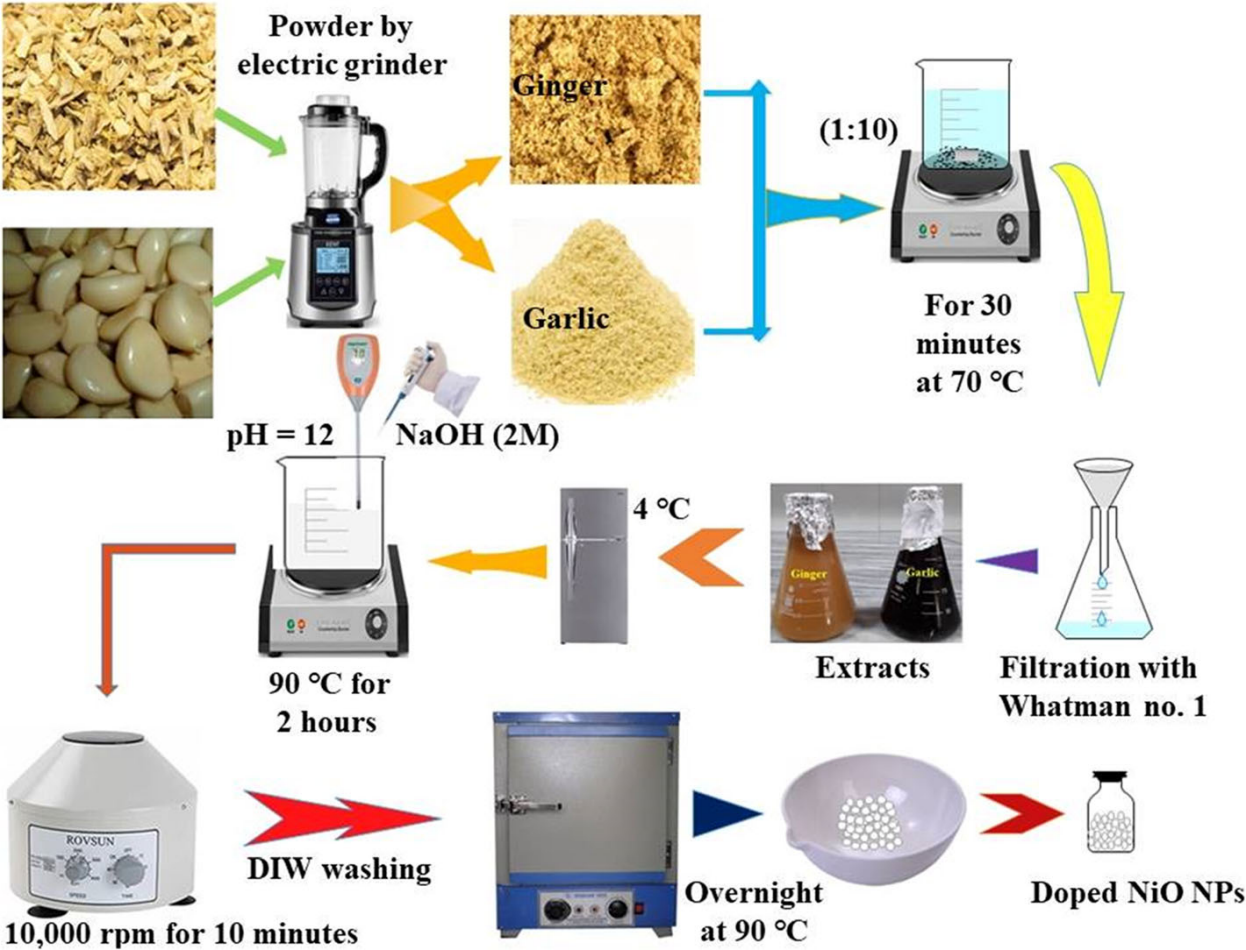
Synthesis scheme of extraction of ginger and garlic and phytochemically reduced NiO-NPs

Ginger and garlic roots were pulverized to fine dust by using electric grinder and preserved in plastic containers. Grounded root’s powder were mixed with controlled quantity of distilled water-DIW (1:10) under vigorous stirring at 70 °C for 30 min. Extracts were cooled, filtered by Whatman No.1 filter paper, and stored at 4 °C (Fig. 1) till further use.

### Green Synthesis of NiO-NPs

Ginger and garlic aqueous extracts of various ratios (1.2, 1.8, 2.4, 3.0, 3.6, and 4.2 ml) were added to nickel nitrate (0.1 M) under continuous stirring. Stirred solution pH 12 was maintained using NaOH (2 M) at 90 °C for 2 h. The precipitates formed were centrifuged at 10,000 rpm for 10 min, washed with DIW, and were dried overnight in hot air oven at 90 °C illustrated in Fig. 1.

### Characterization

Absorption maxima (*ƛ*_max_) of synthesized NPs were scanned by UV-Visible spectrophotometer (Genesys 10 S) from 200–800 nm wavelengths. Phase composition and structural information were analyzed by X-ray diffraction (XRD) BUNKER D2 phaser having 2θ range of (10–80°) equipped with Cu Kα1 radiations of *λ* = 1.540 Å. Green synthesized NiO-NP functional groups were recorded using Fourier-transform infrared spectroscopy (ATR-FTIR). Size, shape, and elemental analysis of NPs were analyzed by field emission electron microscope (FESEM) and transmission electron microscopy Hitachi H7100FA (TEM). The sample composition with corresponding band gaps were investigated by X-ray photoelectron spectroscopy (XPS).

### Isolation and Identification of MDR *S. aureus*

#### Isolation of *S. aureus*

Clinically positive bovine milk samples collected from private and public sector veterinary hospitals and farms in Punjab, Pakistan, were cultured upon 5% sheep blood agar and incubated at 37 °C for 24–48 h. The characteristic colonies obtained were further streaked on mannitol salt agar (MSA) TM Media (Titan Biotech Ltd, India) in triplets to isolate purified *S. aureus*.

#### Identification of MDR *S. aureus*

Identification of bacterial colonies was done through morphological characteristics, Gram’s staining, and biochemical procedures (coagulase and catalase test) as per description of Burgey’s manual of determinative bacteriology.

Antibiotic susceptibility of characteristic colonies was evaluated by disk diffusion test based upon guidelines of National Committee for Clinical Laboratory Standards (NCCLS) for isolation of MDR *S. aureus*. Antibiotic discs containing oxytetracycline (30 μg), tylosine (30 μg), gentamicin (10 μg), ciprofloxacin (5 μg), and trimethoprim + sulphamethoxazole (1.25 μg + 23.75 μg) applied aseptically on Mueller-Hinton agar (MHA) TM Media (Titan Biotech Ltd, India)1 × 10^8^ CFU/ml were kept at 37 °C for 24 h [7]. Bacterium found resistant to minimum three antibiotics was declared MDR [28].

### Antimicrobial Activity

In vitro antimicrobial action potential of phytochemically reduced NiO-NPs was evaluated by agar well diffusion method upon ten representative isolates of MDR *S. aureus* collected from mastitic milk. Petri dishes were swabbed with 1.5 × 10^8^ CFU/ml (0.5 McFarland standard) MDR *S. aureus* on MSA. Wells of 6 mm diameter were formed using sterile cork borer. Various concentrations of individual aqueous extracts of ginger, garlic, and green synthesized (phytochemically reduced) NiO-NPs were applied. Aqueous extracts were used at concentrations of (10 mg/100 μl) and (50 mg/100 μl) and NiO (0.5 mg/50 μl) and (1.0 mg/50 μl). Ciprofloxacin (0.005 mg/50 μl) was used as positive control and DIW as negative control (50 μl).

#### Statistical Analysis

The antimicrobial efficacy was calculated in terms of inhibition zone (mm) size, and inhibition zone diameters were analyzed statistically by one-way analysis of variance (ANOVA) using SPSS 20.

### Catalysis

**Fig. 2:**
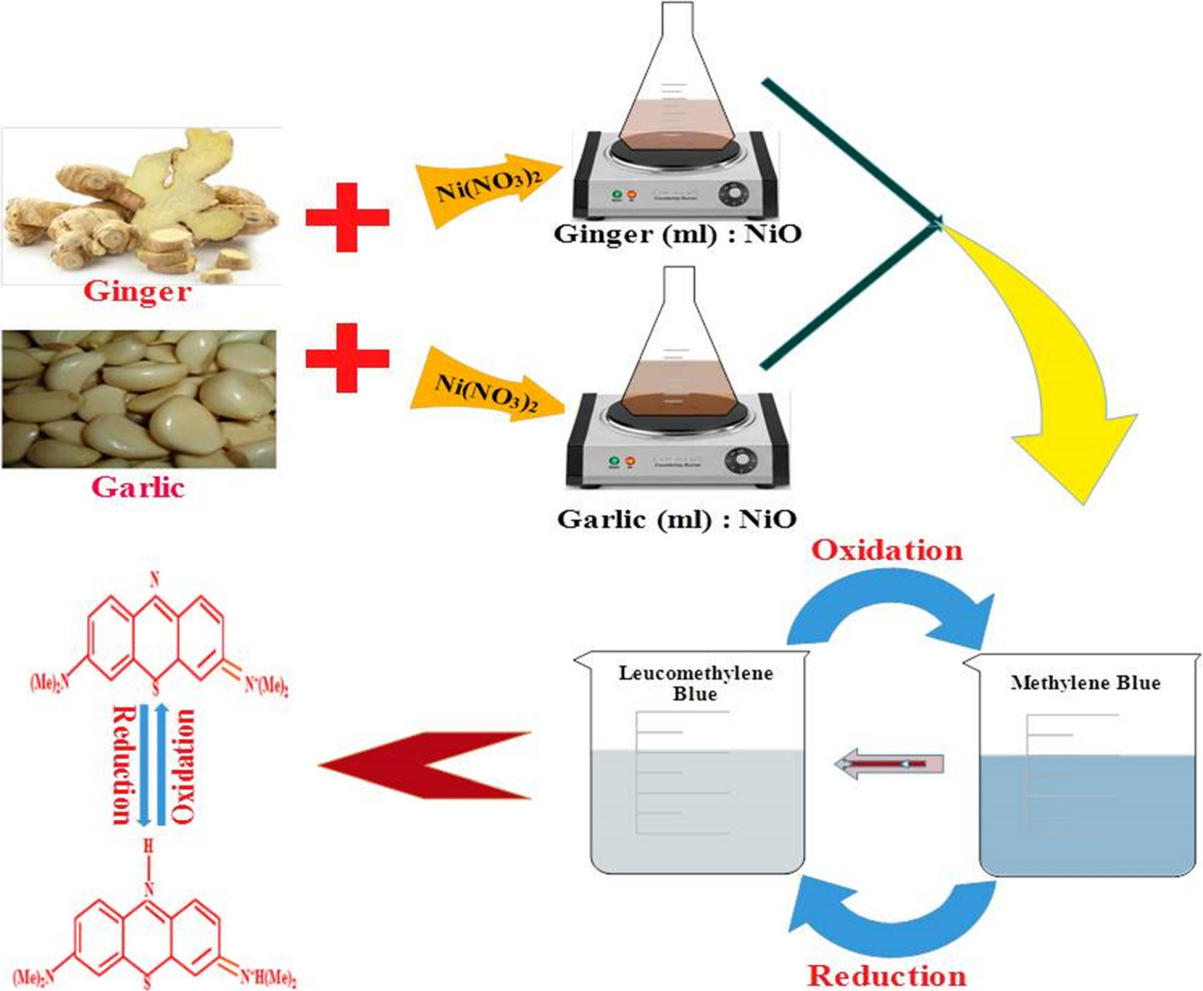
Schematic diagram for catalytic reduction of MB to LMB by green synthesized NPs

For catalytic evaluation of synthesized extract NiO, freshly prepared aqueous sodium borohydride (300 μl) was mixed with 3 ml methylene blue (0*.*03 × 10^−3^ M) solution. Subsequently, 300 μl colloidal sample of desired concentration was added to solutions. Light blue color of methylene blue dye (MB) disappeared representing dye degradation to leucomethylene blue as shown in Fig. 2. The absorption was noted between 200–800 nm using UV-Vis spectrophotometer.

## Results and Discussion

**Fig. 3:**
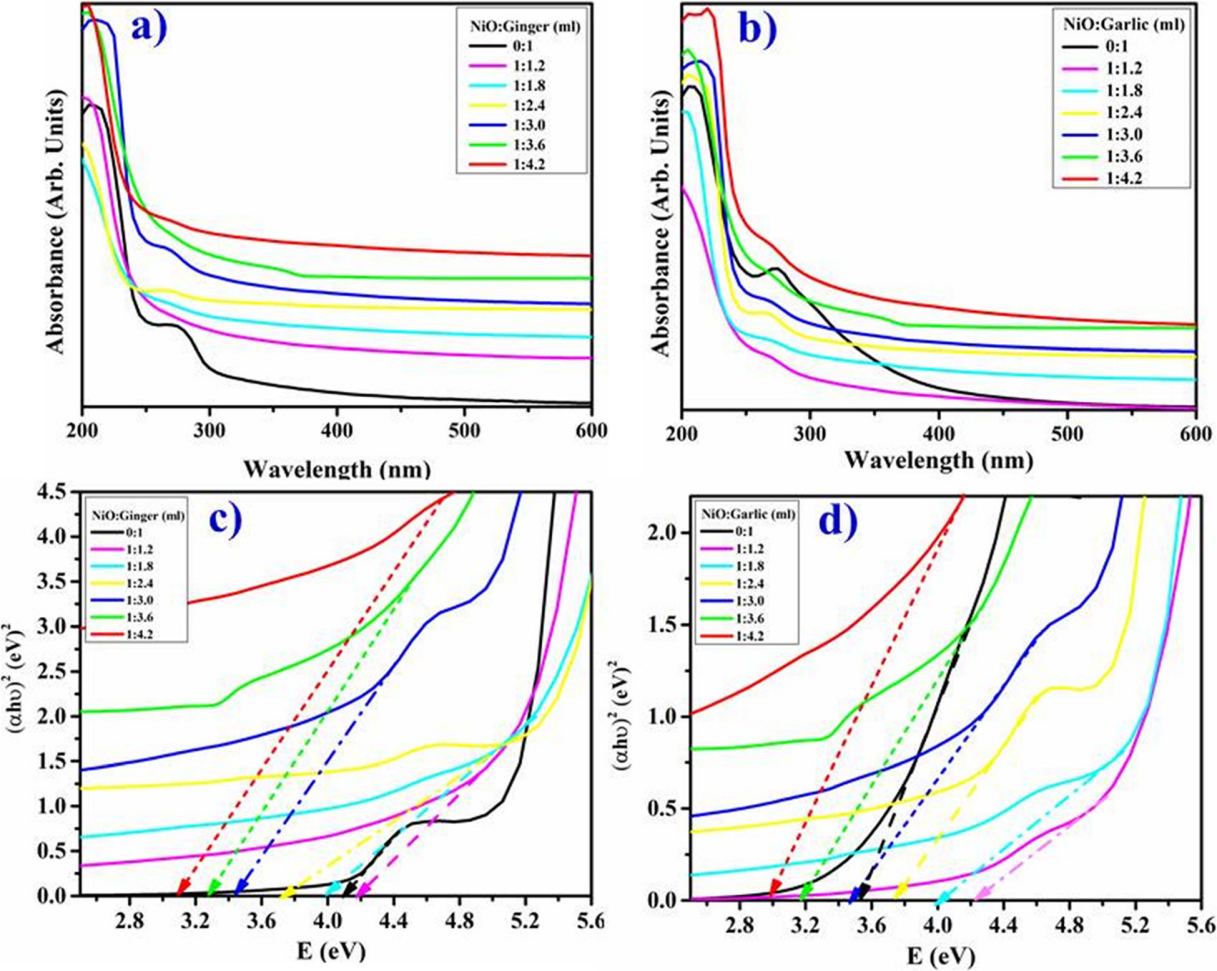
**a**–**d** Absorption spectra of green synthesized NiO-NPs with ginger (**a**) and garlic extract (**b**). Band gap of phytochemically reduced NiO by ginger (**c**) and garlic (**d**) respectively

Optical properties of phytochemically reduced NiO by ginger and garlic aqueous extracts between 200–600 nm are presented in Fig. 3a, b. The maximum absorbance (*λ*_max_) in NiO-NPs was observed around 350 nm (1:3.6 ml) which increased with extracts concentration accompanied by blue shift. Absorption peaks of ginger and garlic extracts appeared around 275 and 280 nm, respectively. Abrupt color change in reaction mixtures was seen from wine red to light green after incorporation of root extracts. Peak broadness indicated particles agglomeration and electronic transition from valence to conduction bands with extract concentration in NiO as revealed by strong absorption bands [20]. Hence, in Fig. 3a, b results showed decrease in absorption of synthesized NPs with increasing or decreasing extract volume beyond optimized value (1:3.6 ml). The band gap was calculated using Tauc’s plot (Eq. 1).

1 $$ \left(\alpha hv\right)=B{\left( hv-{E}_g\right)}^{\raisebox{1ex}{$1$}\!\left/ \!\raisebox{-1ex}{$2$}\right.} $$

Where *α* is the absorption coefficient, *h* is the Planck’s constant, *B* is a constant, *υ* is known as photon frequency, and *E*_g_ is the energy bandgap. The estimated bandgap of phytochemically reduced NiO by ginger and garlic from a plot of (*αhʋ*)^1/2^ against photon energy (*hʋ*). The intercept of a tangent to *x*-axis was recorded, which provides band gap energies of samples as shown in Fig. 3c, d. The variations in band gap energies were determined upon ginger doping into NiO from 4.15 to 3.1 eV and with garlic from 3.5 to 3.0 eV respectively (Fig. 3c, d).

**Fig. 4:**
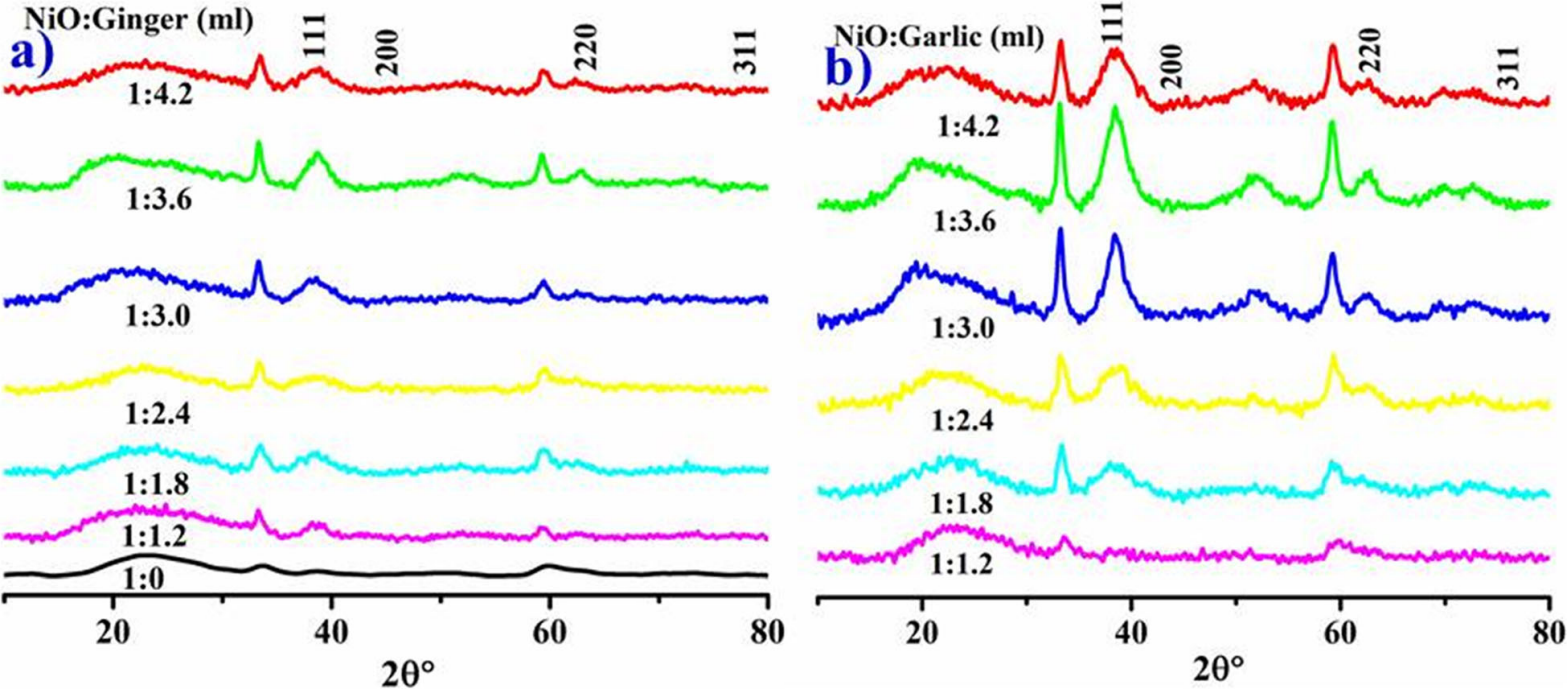
XRD pattern of different concentrations of phytochemically reduced NiO by ginger (**a**) and garlic (**b**) and standard NiO (**c**)

NiO-NPs crystallinity, size, and phase composition were confirmed by XRD as shown in Fig. 4 a, b. The peaks at 2θ values 37.10°, 43.32°, 62.81°, and 76.51°correspond to (111), (200), (220), and (311) (JCPDS card no: 00-047-1049) (Fig. 4a, b) referenced by [30]. The peak intensity indicates hexagonal and face-centered cubic (fcc) NiO with average size 32.9 nm calculated by *D* = 0.9λ/βcosθ for ginger and 29.92 nm for garlic phytochemically reduced NiO-NPs. The broad peaks indicate presence of oxygen spaces and local lattice disorder in sample [38]. Various phytochemicals of ginger (flavonoids, alkaloids, tannins, and saponins) and garlic aqueous extracts (allicin, allyl sulphide, alliin, fatty acids, glycolipids, phenolics, amino acids, and flavonoids) acting as capping agents are responsible for average crystallite size of metal oxide NPs [14, 46].

**Fig. 5:**
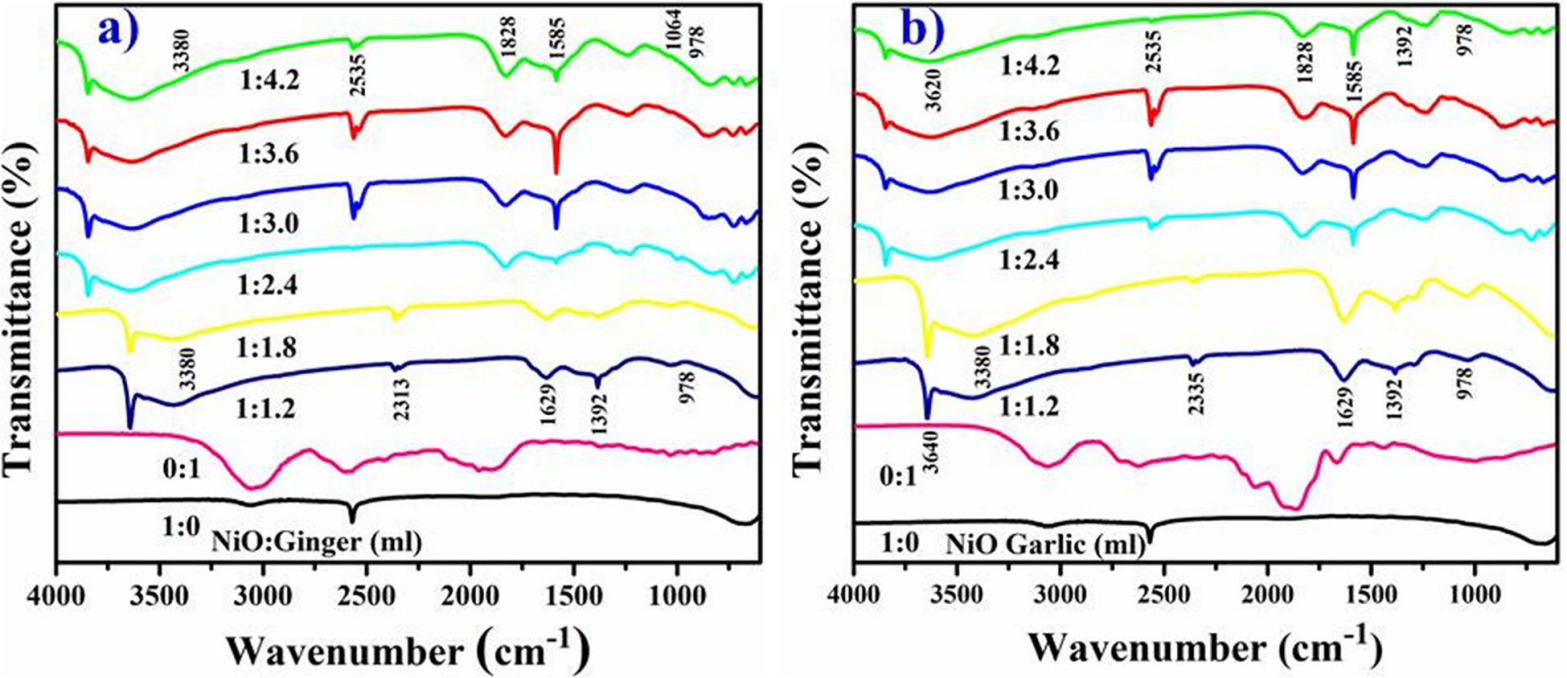
FTIR spectra with ginger extract to NiO (**a**) and garlic (**b**)

The recorded FTIR spectra of NiO biosynthesized from ginger and garlic roots is shown in Fig. 5 a, b. Elaborate broad absorption at 3380 cm^−1^ correspond to OH and peak broadness indicate carbonyl group with (N-H) amine stretching frequency [50]. The sharp absorption at 2313 cm^−1^ indicates stretching vibrations of CO_2_ either aerial or CO_2_ inside NP grains. Rapid absorption of atmospheric CO_2_ indicates greater surface area of material [18]. The broad absorption at 1629 cm^−1^ correspond to C=C aromatic ring stretching and sharp peaks at 1392 and 1064 cm^−1^ correspond to stretching vibrations of C–N aliphatic amines [48]. The intense peaks at 978 cm^−1^ confirmed metal oxygen stretching frequency of NiO [44].

The peak shifts observed after bio-reduction of NiO as 2535–2313, 1828–1629, and 1585–1392 cm^−1^ indicate phytochemicals, terpenoids, flavonoids, polyols, and proteins having ketones, alcohols, carboxylic acid, and amines functional groups responsible for chelating and capping in bio-reduction [42].

**Fig. 6:**
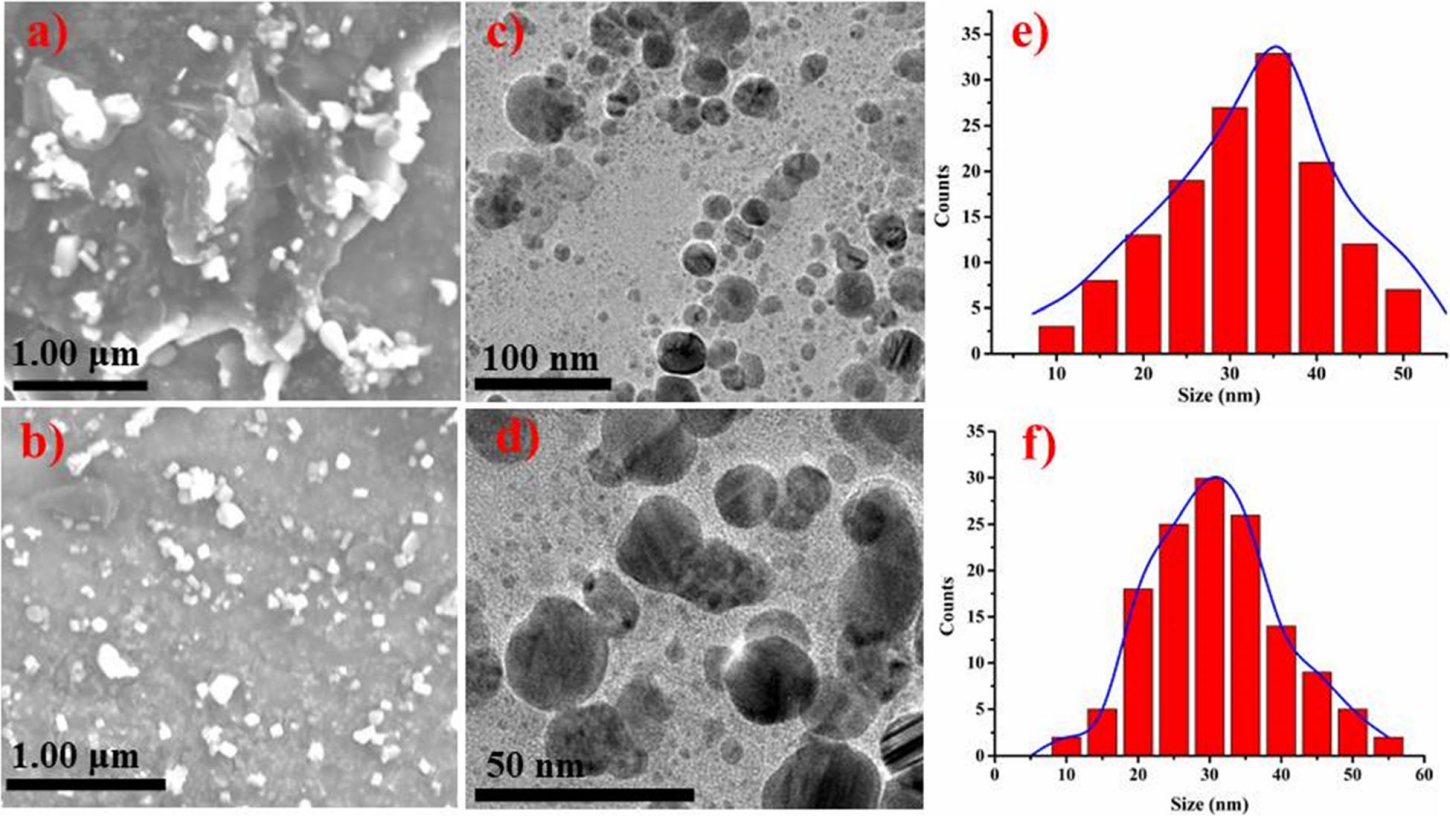
**a**–**f** SEM image of phytochemically reduced NiO by ginger (**a**) and garlic (**b**). TEM image of phytochemically reduced NiO with ginger (**c**) and garlic (**d**) and size distribution of phytochemically reduced NiO by ginger (**e**) and garlic (**f**)

Surface morphology and size of phytochemically reduced NiO-NPs were determined using field emission scanning and transmission electron microscopy as presented in Fig. 6a–f. The NiO-NPs showed pleomorphism with cubical and more spherical shape (< 50 nm) having slight agglomeration [40]. The agglomeration of NPs could be evident from polymer adherence and magnetic interaction between the particles [49].

**Fig. 7:**
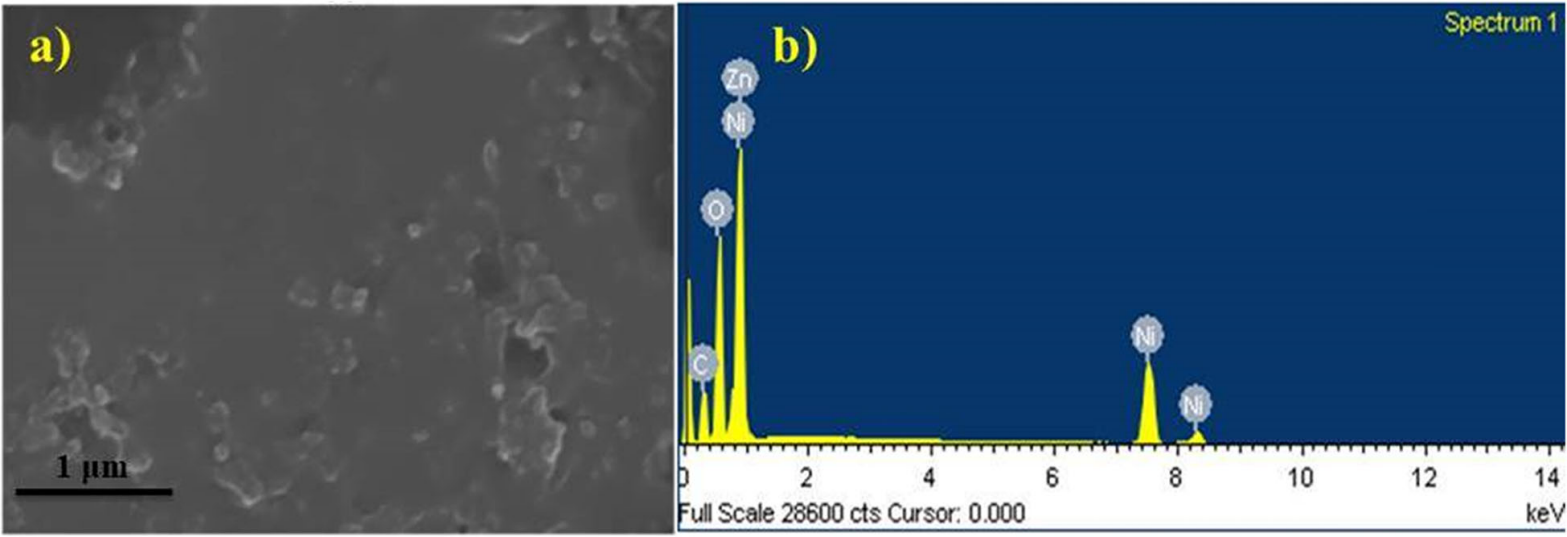
**a**, **b** EDS spectrum of green synthesized NiO-NPs

Elemental analysis and further features of synthesized NiO-NPs were described by energy-dispersive X-ray spectroscopy (EDS) which confirmed pure NiO phases as shown in Fig. 7a, b. The EDS spectra confirmed three peaks directly related to high purity of Ni present in tested samples between 1 and 10 kV. The atomic weight percentages observed through spectra for Ni, O, C, and Zn are 54.69, 27.81, 18.06, and − 0.55, respectively.

**Fig. 8:**
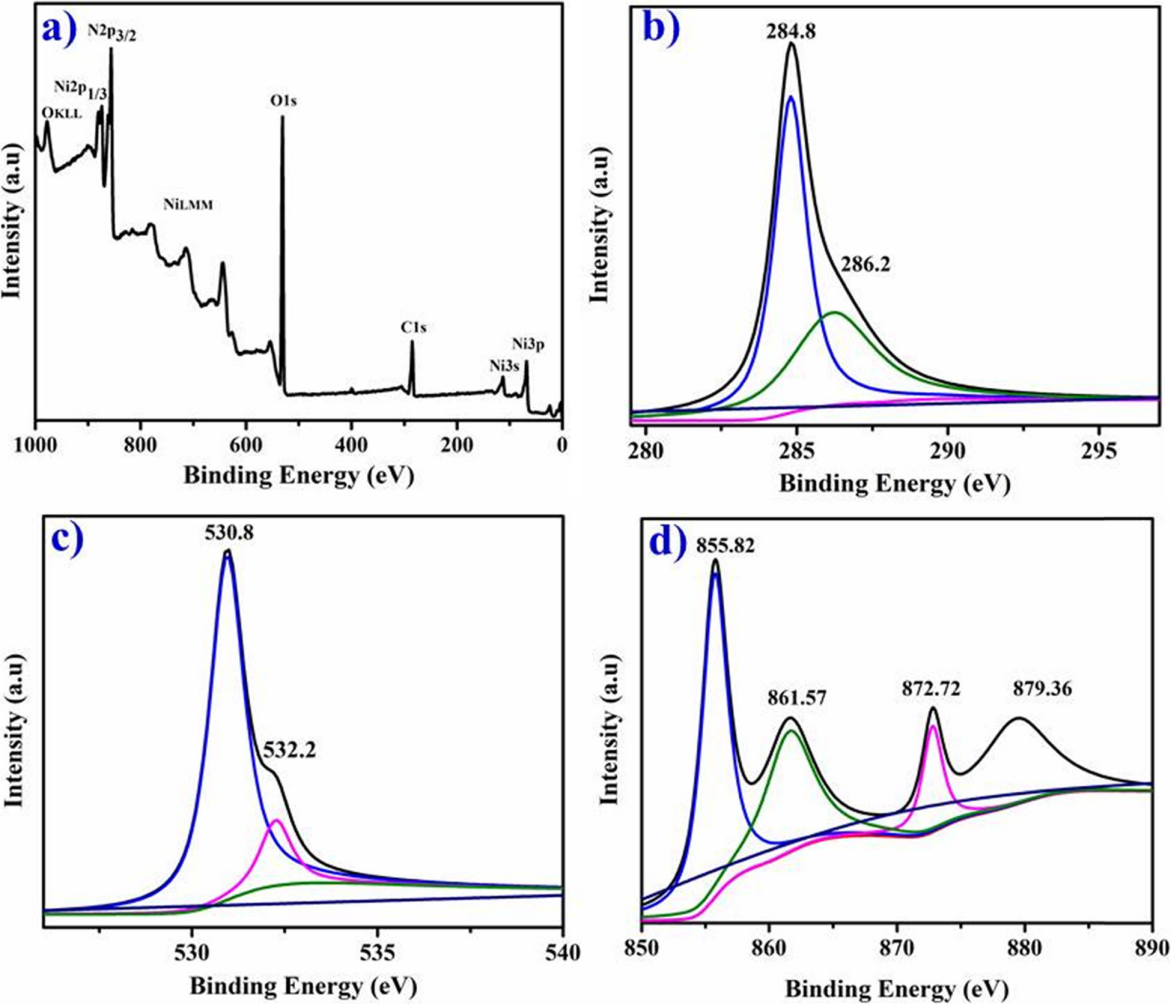
**a**–**d** XPS survey of phytochemically reduced particles (**a**), C1*s* orbitals (**b**), O1*s* spectra of NiO (**c**), and Ni2*p* (**d**)

XPS is showing C1*s*, O1*s*, and Ni2*p* spectra of phytochemically reduced NiO-NPs in Fig. 8a–d that suggests chemical nature and bonding states of synthesized samples. The most intense peaks at 284.8 and 286.2 eV demonstrate C1*s* spectrum (Fig. 8b) corresponding to C–C and C–OH/C–O–C [21]. The O1*s* peak at 530.8 eV (Fig. 8c) could be assigned to hydroxyl groups of oxygen atoms, oxygen atoms adjacent to nickel vacancies, or oxygen-bounded carbon atoms C=O [1, 15, 37]. The contribution located at 532.2 eV ascribes to oxygen atoms in absorbed water molecules (NiOH) [31, 41]. The Ni2*p* spectrum containing Ni2*p*3/2 and Ni2*p*1/2 peaks can be separated using Gaussian–Lorentzian function into five components (Fig. 8d). The most intensive peaks at 872.72 and 855.82 eV belong to Ni2*p*1/2 and Ni2*p*3/2 with corresponding satellite peaks 879.36 and 861.57 eV, respectively [16]. The spin–orbit splitting between the Ni (2*p*1/2) and Ni (2*p*3/2) and NiO-NP core level is 17.28 eV which correspond well with earlier reports [33, 34].

**Fig. 9:**
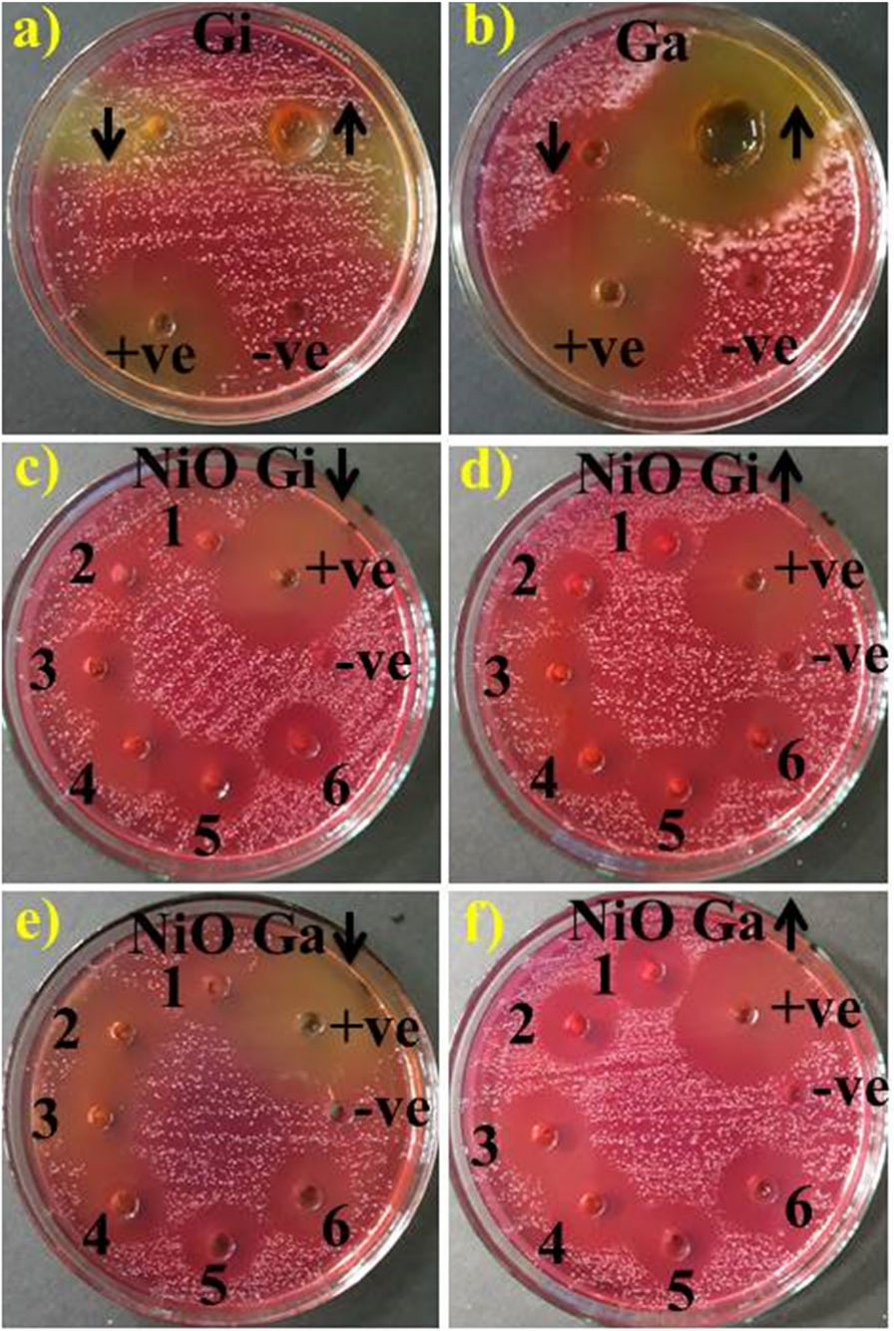
**a**–**f** In vitro antimicrobial activity of ginger aqueous extract (**a**), garlic (**b**), and NiO-NPs phytochemically synthesized by ginger extract at low and high doses (**c**, **d**) and garlic (**e**, **f**)

The antimicrobial/bactericidal action of ginger and garlic root extracts and green/phytochemically reduced NiO-NPs was evaluated using agar well diffusion assay via inhibition zone measurement (mm) as shown in Fig. 9a–d and Table 1. The results depicted strong relation between NP concentration and inhibition zones (mm). Significant inhibition zones (mm) (*P* < 0.05) were recorded for sample 1 (1.2 ml:1), 2 (1.8 ml:1), 3 (2.4 ml:1), 4 (3 ml:1), 5 (3.6 ml:1), and 6 (4.2 ml:1) ranging (3–4.9 mm) and (3.05–5.2 mm) at low and high concentrations phytochemically reduced NiO-NPs by ginger (Fig. 9c, d), while (3.15–5.3 mm) and (3.75–5.9 mm) phytochemically reduced NPs with garlic against MDR *S. aureus* (Fig. 9e, f)*.* Ginger root aqueous extracts depicted zero efficacy (Fig. 9a), and garlic roots showed 2.65 and 5 mm inhibition zones at low and high concentrations, respectively (Fig. 9b). All results were compared to negative control DIW (0 mm) and positive control ciprofloxacin (12.55 mm). Overall, phytochemically reduced NiO-NPs with garlic showed significant (*P* < 0.05) enhanced bactericidal action against MDR *S. aureus.*

**Table 1:** Antimicrobial activity of NiO-NPs

Microorganism	Sample	^1^Inhibition zone (mm)		^2^Inhibition zone (mm)	
		0.5 mg/50 μl	1.0 mg/50 μl	0.5 mg/50 μl	1.0 mg/50 μl
MDR *S. aureus*	(1.2 ml:1) 1	3	3.05	3.15	3.75
	(1.8 ml:1) 2	3.2	3.5	3.4	4.25
	(2.4 ml:1) 3	3.7	3.85	3.9	4.85
	(3.0 ml:1) 4	4.2	4.5	4.35	5.20
	(3.6 ml:1) 5	4.9	5.2	5.3	5.9
	(4.2 ml:1) 6	4.5	4.95	5	5.75
	Ciprofloxacin	12.55	12.55	12.55	12.55
	DIW	0	0	0	0

^1^Inhibition zone of green synthesized NiO from ginger extract

^2^Inhibition zone measurements (mm) of NPs incorporated by garlic extract

The difference in oxidative stress tolerance depends upon various factors as surface area, morphology, and particle size of synthesized nanomaterial playing inferential role in antibacterial action potential [29, 36]. An electrostatic interaction between bacterial strains and nano-scaled materials results generation of reactive oxygen species found responsible for bacterial cell death [2–5, 22]. Two reactions found possible for nanomaterial reaction with bacterial strains including strong interaction of cations Ni^2+^ with bacterial cell negatively charged parts resulting in collapse, while second reaction results in electronic excitation from valance to conduction band upon irradiation of NiO surface with light. Further electronic reaction with O_2_ generates O^−^_2_ radicals resulting in H_2_O_2_ production. The ·OH production occurred upon reaction of h^+^ with water. Thus, resulting O^−^_2_· and ·OH species played significant role in breaking down of lipid or protein molecule present in bacterial cell outer surface [39].

### Catalytic Activity

**Fig. 10:**
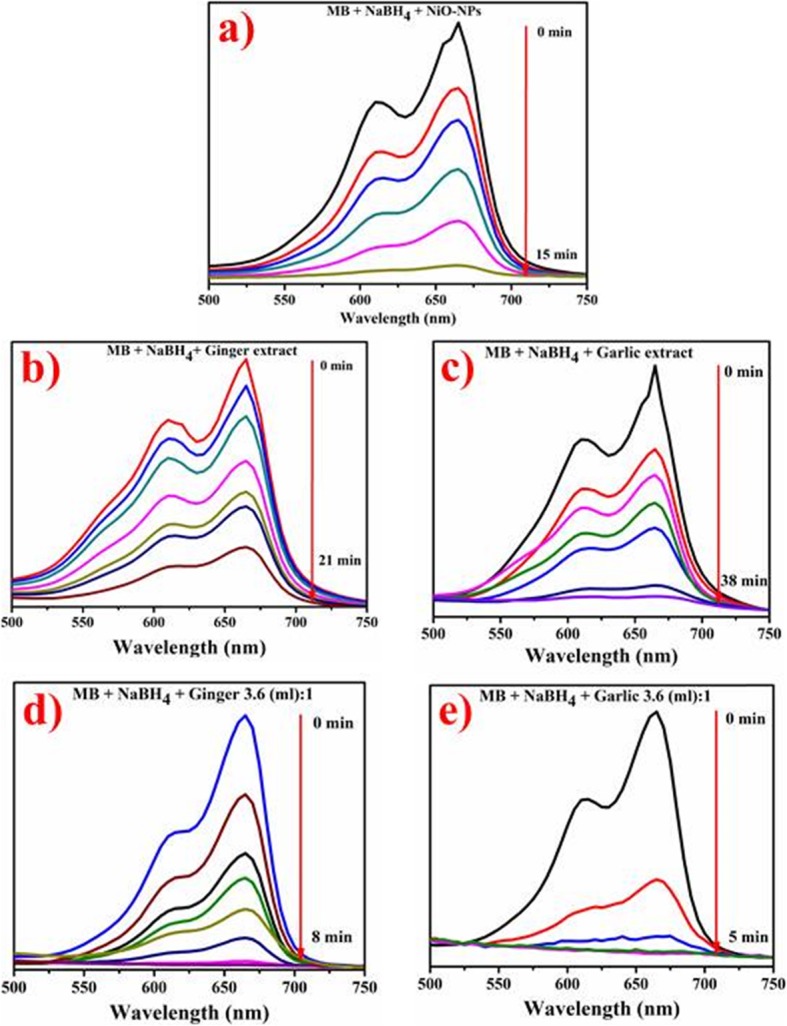
**a**–**e** Catalytic activity of NiO (**a**), ginger extract (**b**), garlic extract (**c**), phytochemically reduced NiO by ginger (**d**), and garlic reduced NPs (**e**)

Figure 10 a–e demonstrating MB catalytic reduction in the presence of root extracts and green/phytochemically reduced NiO-NPs at room temperature. Figure 10 a shows catalytic potential of NiO-NPs synthesized by conventional route while (Fig. 10b, c) represents catalytic potential of ginger and garlic root aqueous extracts. Catalytic capacity of phytochemically reduced NiO-NPs is represented in Fig. 10d, e. It is obvious that NiO and plant root extracts are not an efficient nano-catalyst as they were consuming 15, 21, and 38 min for methylene blue reduction (Fig. 10a–c). Phytochemically reduced NPs with ginger showed quick degradation (*λ*_max_ = 8 min) with efficient conversion of MB to leucomethylene blue (Fig. 10d). Garlic mediated NiO-NPs showed similar pattern of 100% dye reduction in 5 min (Fig. 10e).

Green/phytochemically reduced NPs perform significant catalytic dye degradation by transferring electrons from donor species (BH_4_) to acceptor (MB) and stabilize system by reducing activation energy [27]. The data revealed green NPs as efficient nano-catalyst compared to conventional NPs and individual extract.

## Conclusion

NiO-NPs having ginger and garlic root extracts served as excellent bactericidal as well as catalytic agent. Root extract incorporation having phytochemical groups resulted in successful NiO-NP synthesis revealed by FTIR. The XRD peaks confirmed NiO hexagonal and face-centered cubic (fcc) lattice and SEM confirmed pleomorphism with cubical and more spherical morphology of NPs having average size 16–52 (ginger doping) and 11–59 nm (garlic doping). However, elemental analysis revealed chemical nature and bonding states analyzed by EDS and XPS and presented actual percentage of nickel and oxygen, while UV analysis verified absorption peaks difference in range 350 nm and introduced blue shift at higher amount of dopants. Phytochemically garlic reduced NiO at high concentration was found more potent compared to ginger reduced NPs against MDR *S. aureus* as well as reduced MB efficiently. Thus, green/phytochemically reduced NiO from garlic root extracts may be adopted in advanced medicine as substitute of antibiotic resistance and in textile industries as catalytic agent with no environment hazard.

## Data Availability

All data are fully available without restriction.

## Abbreviations

EDS: Energy-dispersive X-ray spectroscopy
fcc: Face-centered cubic
FTIR: Fourier-transform infrared spectroscopy
G +ve: Gram positive
G –ve: Gram negative
JCPDS: Joint committee on powder diffraction standards
MB: Methylene blue
Ni: Nickel
NiO: Nickel oxide
nm: Nanometer
NPs: Nanoparticles
SEM: Scanning electron microscopy
TEM: Transmission electron microscopy
UV-Vis: Ultra-violet visible spectroscopy
XPS: X-ray photoelectron spectroscopy
XRD: X-ray diffraction
